# Benefits of Implicit Regulation of Instructed Fear: Evidence From Neuroimaging and Functional Connectivity

**DOI:** 10.3389/fnins.2020.00201

**Published:** 2020-03-13

**Authors:** Yicheng Zhang, Shengdong Chen, Zhongyan Deng, Jiemin Yang, Jiajin Yuan

**Affiliations:** ^1^The Laboratory for Affect Cognition and Regulation (ACRLab), Faculty of Psychology, Southwest University, Chongqing, China; ^2^Institute of Brain and Psychological Sciences, Sichuan Normal University, Chengdu, China

**Keywords:** implicit emotion regulation, instructed fear, fMRI, functional connectivity, amygdala

## Abstract

Instructed fear, which denotes fearful emotions learned from others’ verbal instructions, is an important form of fear acquisition in humans. Maladaptive instructed fear produces detrimental effects on health, but little is known about performing an efficient regulation of instructed fear and its underlying neural substrates. To address this question, 26 subjects performed an instructed fear task where emotional experiences and functional neuroimages were recorded during watching, explicit regulation (calmness imagination), and implicit regulation (calmness priming) conditions. Results indicated that implicit regulation decreased activity in the left amygdala and left insula for instructed fear; however, these effects were absent in explicit regulation. The implementation of implicit regulation did not increase activity in the frontoparietal control regions, while explicit regulation increased dorsolateral prefrontal cortex activity. Furthermore, implicit regulation increased functional connectivity between the right amygdala and right fusiform gyrus, and decreased functional connectivity between the right medial temporal gyrus and left inferior frontal gyrus, which are key nodes of memory retrieval and cognitive control networks, respectively. These findings suggest a favourable effect of implicit regulation on instructed fear, which is subserved by less involvement of control-related brain mechanisms.

## Introduction

‘Nian’ is a ferocious monster in a Chinese fairy tale, which is said to devour people in the evening before the Spring Festival. Although no one has seen the Nian, the ancient Chinese fear Nian so much, that they set off firecrackers to drive it away on the eve of the Spring Festival. Such fear learned from the verbal instruction of threat from others, is termed instructed fear (Phelps et al., 2001; Olsson and Phelps, 2007). As an important form of social learning of fear, the instructional learning of fear can be a robust and stable cause of fear (Koban et al., 2017). Olsson and Phelps (2007) demonstrated that instructional pathways to fear can also have strong effects compared to those of other pathways to fear (e.g., by classical conditioning or observation).

Maladaptive instructed fear produces detrimental effects on health, such as distorting environmental perception and disruption of normal functioning (Olsson and Phelps, 2007) or generating excessive fear associated with phobic and post-traumatic stress disorders (Etkin and Wager, 2007; Jovanovic et al., 2010). Instructed fear also produces long-lasting emotional impact, resistance to the extinction procedure, and contributes to the development of anxiety disorders (Field and Storksen-Coulson, 2007; Bublatzky et al., 2014). However, little is known about the regulation of instructed fear and its neural underpinnings.

Emotion regulation is critical to avoid the adverse effects of abnormal fear, which can be realized by both explicit and implicit processes (Gyurak et al., 2011). The explicit form of emotion regulation is implemented with explicit regulatory goals. For example, Delgado et al. (2008) instructed participants to regulate their conditioned fear responses by imagining something calming in nature during emotion regulation trials, which successfully diminished fear responses but increased cognitive cost. Although the effect of explicit imagination on conditioned fear has been demonstrated, the effectiveness of this strategy relative to that of regulation of instructed fear learning remains unknown. Implicit emotion regulation can be defined as a process that aims to modify the quality, intensity, or duration of an emotional response without the need for conscious supervision and explicit intentions, thus requiring little cognitive cost (Koole and Rothermund, 2011). Implicit emotion regulation is often realized through unconscious goal pursuit. For example, Yang et al. (2014) found that priming emotion regulation reduced emotional responses to both gains and losses without the cost of cognitive resources. In order to identify an appropriate form of emotion regulation for instructed fear, we aimed to investigate the regulatory effects and cognitive cost of explicit and implicit emotion regulation on instructed fear using a classic instructed fear paradigm (Phelps et al., 2001; Olsson and Phelps, 2007).

Previous functional magnetic resonance imaging (fMRI) studies have shown that the amygdala and insula are key regions involved in the social learning of fear, including both observational and instructional forms of fear acquisition (Funayama et al., 2001; Phelps et al., 2001; Mechias et al., 2010; Maier et al., 2014; McMenamin et al., 2014). Phelps et al. (2001) observed an enhanced activity in the left amygdala in a threat condition relative to a security condition using the instructed fear paradigm, suggesting that one function of the amygdala is the expression of instructed fear. Researchers also suggest that the amygdala is a key brain region mediating social learning of fear and maintaining vigilance to potential danger (Funayama et al., 2001; Phelps et al., 2001; Butler et al., 2007; Mechias et al., 2010). Moreover, the insular cortex is considered to convey the cortical representation of social learning of fear to the amygdala (Phelps et al., 2001; Butler et al., 2007) and is implicated in anticipation of harmful stimuli (Olsson and Phelps, 2007). Previous research on conditioned fear has consistently demonstrated that the amygdala and insula are robustly activated when watching a threat-conditioned stimulus (CS+) relative to a safety-conditioned stimulus (CS−) (Etkin and Wager, 2007; Olsson and Phelps, 2007; Salomons et al., 2007; Delgado et al., 2008; Sarinopoulos et al., 2009; Ma et al., 2013). Decreased activation of the amygdala and insula is associated with reduced negative emotion (Delgado et al., 2008; Goldin et al., 2008). These findings indicate that the amygdala and insula are essential neural substrates mediating fear elicitation and extinction, irrespective of how fear is elicited. Thus, amygdala and insula activity should be used to evaluate the intensity and regulation of instructed fear responses.

In this study, we used a calmness imagination task and a synonym matching task to initiate explicit emotion regulation (EER) and implicit emotion regulation (IER), respectively. The explicit and IER paradigms have proven effective in decreasing amygdala activity linked with fear-conditioned stimuli (Delgado et al., 2008) and in reducing the electrophysiological responses to gains and losses (Yang et al., 2014), respectively. In order to compare the cognitive cost of regulating fear explicitly and implicitly, we examined differences in activity changes in the typical frontoparietal cognitive control regions, including the dorsolateral prefrontal cortex (dlPFC), dorsal anterior cingulate cortex (dACC), and inferior parietal lobule (IPL) (Ochsner et al., 2002; Lewis and Miall, 2003; Harvey et al., 2005; Dosenbach et al., 2008; Goldin et al., 2008; Vincent et al., 2008; Niendam et al., 2012; Power and Petersen, 2013).

Research has demonstrated that imagination strategies are effective for emotion regulation (Wolpe, 1961; Kalisch et al., 2006; Delgado et al., 2008; Goldin et al., 2008). However, explicit imagination is an effortful strategy, whose execution comes at the cost of increasing cognitive load (Ochsner et al., 2002; Goldin et al., 2008; Hutcherson et al., 2012). Therefore, we predicted that the implementation of EER might be accompanied by substantial involvement of cognitive control regions. By contrast, IER has proven valid in reducing stress or frustration-related physiological activity (Mauss et al., 2007; Eder, 2011; Yuan et al., 2015a) without maladaptive cardiovascular consequences or cognitive resource depletion (Bonanno et al., 2004; Mauss et al., 2007; Fiori, 2009; Gyurak et al., 2011). Based on these findings, we predicted that IER may decrease activity in the amygdala and insula without increasing activity in cognitive control regions (e.g., dlPFC, ACC, and IPL), a beneficial effect most likely absent during EER. Moreover, we conducted brain network analyses to explore the neural mechanisms underlying the emotion regulation effects of IER. Specifically, according to the evidence reviewed above, we predicted that functional coupling subserving cognitive control that is centred in the prefrontal cortex may be enhanced during EER, an effect that should be absent during IER.

## Materials and Methods

### Participants

We determined the sample size based on *a priori* power analysis using G-power software (Faul et al., 2009). We specified a moderate effect size of 0.25, as reported in related IER research by Tupak et al. (2014), statistical power set at 0.8 to 0.9, and a moderate correlation (0.5) among the repeated measurements, which yielded a recommended sample size of 19–24. Thus, we recruited 26 right-handed college students who were paid to participate in the study (15 males; average age = 20.91 years). All participants were instructed to complete the State-trait Anxiety Inventory (Spielberger, 1983) and Beck Depression Inventory (Beck et al., 1961). Three participants were removed from data analysis because they doubted the truth of the instructions (two) or reported anxiety and depression (one). The remaining 23 participants (12 males; average age = 20.96 years, *SE* = 0.350) had normal vision with or without correction; reported no history of psychiatric disorders, medical disorders, or medication use; and provided written informed consent. This study was approved by the local ethical committee of Southwest University for human brain research. The experimental procedure was in accordance with the ethical principles of the 1964 Declaration of Helsinki.

### Experimental Materials

IER was primed by the Synonym Matching Task (SMT), as this task has been verified to successfully prime IER (Yang et al., 2014). To attribute the emotion regulation effect in the implicit condition to idioms of calmness priming rather than to SMT itself, the SMT with neutral idioms was also used in the control conditions. In total, 54 Chinese four-character idioms were included in the SMT and were classified into two categories according to their meaning, i.e., emotion regulation and neutral idioms. The emotion regulation idioms included 12 idioms that were selected from popular Chinese sayings. These idioms either advised people to keep calm in the face of any consequence or calm down by accepting irrevocable outcomes (e.g., 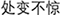, which means keeping calm in an emergency). The neutral idioms were unrelated to emotion regulation (e.g., 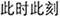, which means right now). The 54 idioms thus included 6 pairs of calmness-related synonyms, 12 pairs of neutral synonyms, and 18 distracting idioms, which formed 6 calmness-related SMT trials, and 12 neutral SMT trials. Three runs were conducted. Each run included two emotion regulation blocks (explicit and implicit) and one watching block. Each block included four threat and four safety trials. Each implicit regulation, explicit regulation, or watching block started with two SMT trials related or unrelated to calmness priming, respectively. Idioms used in SMT were not repeated to avoid habituation during this experiment.

All 54 idioms were evaluated using a 9-point scale by an independent sample of subjects (14 females, 7 males; mean age 24 ± 2.1 years), for valence (1 = extremely negative to 5 = neutral to 9 = extremely positive), arousal (1 = extremely calm to 9 = extremely exciting), and familiarity (1 = extremely unfamiliar to 9 = extremely familiar) dimensions. An independent-sample *t*-test revealed that there were no significant differences between the emotion regulation and neutral idioms in the three dimensions [valence: 6.64 ± 0.99 vs. 5.94 ± 1.27, *t*(52) = 1.75, *p* = 0.09; arousal: 5.46 ± 0.80 vs. 5.87 ± 0.67, *t*(52) = −1.75, *p* = 0.09; familiarity: 7.47 ± 0.63 vs. 7.51 ± 0.42, *t*(52) = −0.3, *p* = 0.77].

### Experimental Procedure and Design

A classic instructed fear paradigm was used to evoke socially instructed fear (Phelps et al., 2001; Olsson and Phelps, 2004, 2007; Butler et al., 2007). After subjects lay in an MRI scanner, electrodes were attached to their left wrist. In order to convince participants that shocks might occur in the experiment, we used electric shock equipment (Inui et al., 2002, 2005) (intraepidermal electrical stimuli, IES) to test the maximum intensity of the shock that participants could stand. Participants were informed that the electrode attached to their wrist would be used to deliver the maximum electric shocks one to three times during the threat condition. Two coloured squares were used as the conditioned threat or safety signal, respectively. For instance, the presentation of a blue square signalled potential shock, thus serving as the CS +, while yellow square presentation signalled safety, thus serving as the CS–. The colours representing threat and safety were counterbalanced across subjects. Although participants believed they would occasionally be shocked, neither CS+ nor CS− was paired with a shock throughout the experiment to avoid a learning effect.

**FIGURE 1:**
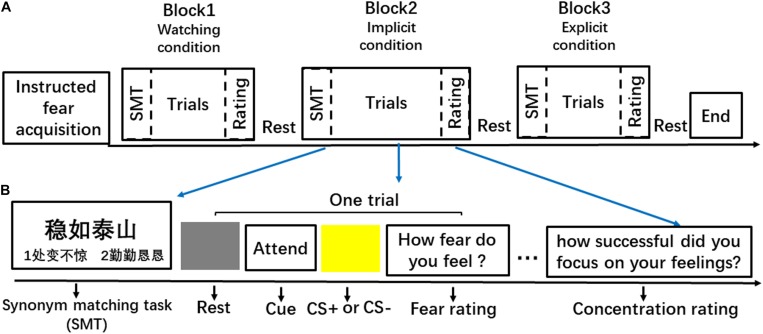
Schematic illustration of the experimental streamline. **(A)** An example of the event organisation in a run. **(B)** An example of behavioural procedure of a block.

A mixed fMRI design was used to induce instructed fear and to assess the regulatory effects of explicit and implicit regulation strategies (Figure 1A). The experiment included three 8-min runs. Each run consisted of three blocks, i.e., two emotion regulation blocks (explicit and implicit) and one watching block. Blocks were randomly intermixed during the presentation. Each block included four threat and four safety trials. Trial order within each block was pseudo-randomized. This experiment consisted of 72 instructed threat or safety trials in total.

Each block (Figure 1B) began with two SMT trials that primed participants with either a goal of emotion regulation (calmness) or neutral concepts. Specifically, the IER block was always paired with calmness-related priming, and the other two conditions were paired with neutral concepts. In the SMT task, participants saw a target idiom at the top of the computer screen and two probe idioms at the left and right side of the bottom of the screen. Subjects had 4 s to indicate which one of the two probe idioms was the synonym of the target idiom by pressing buttons (1 = left and 2 = right). Half of the matching idioms were presented on the right side of the screen, and half of them were presented on the left side.

After the SMT trials, a 2-s word cue was presented before the safety or threat trials in each block. The cues reminded participants of the task to either ‘ATTEND’ or ‘IMAGINE.’ The ‘IMAGINE’ cue was always presented in the explicit regulation blocks, informing participants to imagine something in nature when viewing the CS. For example, participants could think of an image of the ocean or a blue sky when viewing the blue square, and they could think of canola flower fields when viewing the yellow square. In implicit and watching blocks, the ‘ATTEND’ cue instructed the participants to attend to their natural feelings to the presented CS. Then, a blue or yellow square, which signalled threat or safety, respectively, was presented for 4 s in the centre of a grey background. The inter-trial interval (ITI) varied among 6, 8, and 10 s. Each trial concluded with a 4-s scale, which required participants to rate their experienced fear on a 7-point scale (1 = not fearful at all; 7 = extremely fearful). Each block concluded with a 4-s scale requiring participants to assess ‘how they attended to their feelings to the stimulus’ (watching or implicit regulation conditions) or ‘how successful was the imagination’ (explicit regulation condition) during the square presentation using a 7-point scale (1 = not at all; 7 = fully attentive/successful).

### fMRI Acquisition and Analysis

Brain imaging data were acquired with a Siemens 3T scanner (Siemens Magnetom Trio TIM, Erlangen, Germany). Anatomical images were collected with a T1-weighted protocol (TR = 1,900 ms, TE = 2.52 ms, FA = 9°, matrix = 64 × 64, FOV = 256 × 256 mm^2^, voxel size = 1 × 1 × 1 mm^3^). The fMRI images were collected with an echo-planar imaging (EPI) sequence (TR = 2 s, TE = 30 ms, flip angle = 75°, matrix size = 64 × 64, FOV = 220 × 220 mm^2^, voxel size = 3.4 × 3.4 × 3 mm^3^, slices = 32). Before the scanning, all subjects were instructed to remain still and motionless during fMRI scanning. Stimulus presentation and behavioural data acquisition were obtained using E-prime (1.0) software.

Each functional run was subjected to preprocessing steps using DPABI (Yan and Zang, 2010) software: slice-timing, realignment, normalizing to MNI space using with the structure information from coregistration, and segmentation and spatial smoothing with a Gaussian kernel (8 mm FWHM).

Statistical analysis of the preprocessed functional data was performed in statistical parametric mapping (SPM8)^1^ and custom-written programs in Matlab. In the first-level analysis, the three functional scanning runs were modelled in one general linear model. Six periods of interest (‘watch’ CS+, ‘watch’ CS-, ‘implicit’ CS+, ‘implicit’ CS−, ‘explicit’ CS+, and ‘explicit’ CS−) were included in the model to compute linear contrast maps. Six realignment parameters were further included as regressors of no interest to account for head motion effects. The resulted design matrix was then filtered with a high-pass of 128 s.

The major goal of this fMRI study was to characterize the response profile of the implicit and EER in fear-generative and cognitive control-related regions. To this end, region of interest analysis (ROI) was next conducted. Given our *a priori* hypothesis regarding increased activity in the amygdala and insula during the contrast threat versus safety, we set the amygdala and insula as the ROIs. ROI analyses of the amygdala (Ball et al., 2007; Kim and Whalen, 2009) and insula (Semendeferi and Damasio, 2000; Craig and Craig, 2009; Chang et al., 2012) were defined anatomically based on Anatomical Automatic Labeling (Tzourio-Mazoyer et al., 2002).

We also tested whether the activity of cognitive control-related regions varied with IER and EER. The ROIs of the inferior parietal lobule (IPL) were defined using the anatomy toolbox in SPM8 (Eickhoff et al., 2005; Wang et al., 2015). The dlPFC was defined by a mask of Brodmann areas 9 and 46 (Fink et al., 1999; Ragland et al., 2012). For dACC, a sphere ROI was defined based on the coordinates (Talairach coordinates: *x* = 0, *y* = 12, *z* = 42) from a prior meta-analysis (Shackman et al., 2011) using a 10 mm radius. Percent signal change (PSC) for each ROI was then extracted using MarsBaR (Brett et al., 2002).

The emotional effects of instructed fear were operationally defined by the behavioural or PSC contrast values (Garfinkel et al., 2014) (CS+ versus CS−). We then compared emotional effects in four regions (bilateral amygdala and bilateral insula) and the effects of cognitive cost in five regions (bilateral dlPFC, bilateral IPL, and dACC) in respective ROIs across the three conditions (watching, implicit, and explicit). All *p*-values were adjusted with Bonferroni–Holm method (Holm, 1979; Benjamini and Yekutieli, 2001; Storey, 2003; Storey and Tibshirani, 2003; Penders et al., 2010).

We used the CONN toolbox (version 16)^2^ in MATLAB to perform task-related functional connectivity (FC) across the three conditions. There were 229 ROIs included in our analysis: 227 of them belonged to the networks divided by Power and Petersen (2013), and the remaining two were the amygdala and insula defined anatomically based on Anatomical Automatic Labeling (Tzourio-Mazoyer et al., 2002). These 227 ROIs were assigned to several functional networks, comprising low-level input and output networks (visual, auditory, and sensorimotor networks), subcortical nodes, the default mode network (DMN), ventral and dorsal attention networks (VAN and DAN), and cognitive control networks (frontal-parietal network, FPN; cingulo-opercular network, CON; salience network, SN) (Cole et al., 2013.; Mohr et al., 2016). Regional time series within each of these 229 ROIs were extracted from the preprocessed fMRI data at an individual level. The task onset times were modelled, and covariates of no interest were regressed out using a component-based noise correction method (CompCor) (Behzadi et al., 2007).

Time series of voxels within 229 ROIs were averaged, and those average time series were correlated with each other. The resulting correlation coefficients were Fisher *z*-transformed to normalize their distribution. The computed ROI-to-ROI connectivity matrices of each participant were entered into the second-level group analysis via a 3-by-2 ANOVA. False positives in this network analysis were controlled by false discovery rate (FDR) of *P* < 0.05. Based on the survival of 3-by-2 interactions, planned comparisons for each FC were conducted by testing how the FC intensity differences in threat relative to safety trials varied among watching, explicit, or implicit regulation conditions.

## Results

### Manipulation Check

**FIGURE 2:**
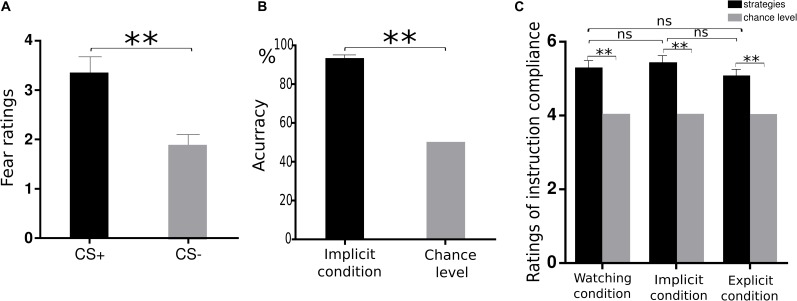
Behavioural data. **(A)** Mean ratings of negative experience for CS+ and CS− trials during watching condition. **(B)** The accuracy in the synonym matching task was significantly higher than chance levels during the implicit regulation condition. **(C)** Similar ratings of instruction compliance across three conditions. **p < 0.01, n.s. denotes not significant.

**FIGURE 3:**
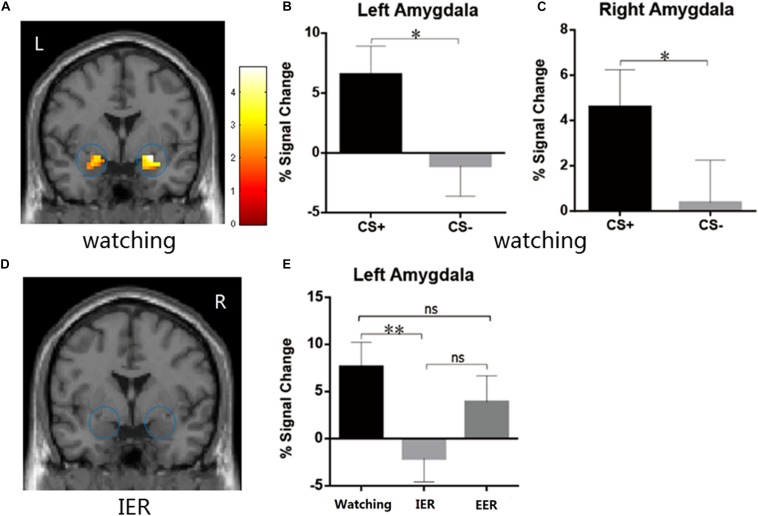
**(A–C)** The watching condition showed higher BOLD signal changes during CS+ relative to CS– in the left and the right amygdala. **(D)** The IER conditions showed similar BOLD responses in the bilateral amygdala during CS+ vs. CS–. **(E)** The IER but not EER demonstrated significantly reduced PSC values (CS+ minus CS–) in the left amygdala relative to that for the watching condition. Error bars = SEM, *p < 0.05, **p < 0.01, n.s. denotes not significant.

**FIGURE 4:**
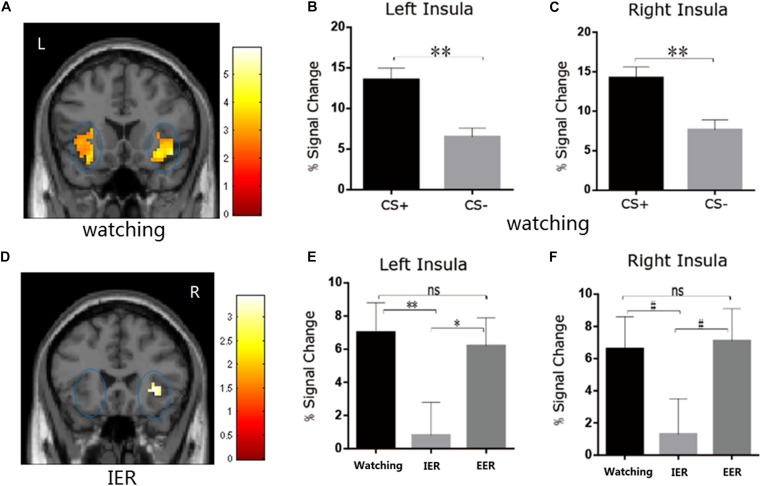
**(A–C)** The watching condition showed higher BOLD signal changes in the left and right insula during CS+ vs. CS– trials. **(D)** The IER condition showed similar BOLD responses in bilateral insula during CS+ vs. CS– trials. **(E,F)** The comparison of BOLD signal changes across watching, IER, and EER conditions in the left and right insula. Error bars denote SEM, *p < 0.05, **p < 0.01, #0.05 < *p* ≤ 0.07; n.s., not significant.

We first examined whether the instructed fear paradigm successfully induced fear emotion. At the behavioural level, we conducted a paired-samples t-test of emotional experience between CS+ and CS− in the watching condition, where emotion effects were free of regulatory influences. The CS+ (*M* = 3.33, *SE* = 0.36) vs. CS− [*M* = 1.89, *SE* = 0.20, *t*(22) = 4.81, *p* < 0.001, Figure 2A] contrast was significant, and all subjects reported feeling greater fear during CS+ trials. At PSC, we observed significant CS+ relative to CS− differences in bilateral amygdala [left: *t*(22) = 2.89, *p* = 0.016, Figures 3A,B; right: *t*(22) = 2.09, *p* = 0.048, Figures 3A,C] and bilateral insula areas with greater activity [left: *t*(22) = 3.96, *p* = 0.009, Figures 4A,B; right: *t*(22) = 3.34, *p* = 0.004, Figures 4A,C] in ‘attend’ CS+ than in ‘attend’ CS−. These results consistently indicated that the instructed fear paradigm successfully induced fear at both experiential and physiological levels.

The second manipulation check aimed to examine whether subjects successfully accessed calmness-related information by critical SMT. We calculated the accuracy of synonym matching in the implicit regulation condition. The accuracy versus chance level of 50% contrast was highly significant [0.92 vs. 0.5, *t*(22) = 18.18, *p* < 0.001, Figure 2B], confirming that the priming of calmness-related meanings was successful.

The third manipulation check examined how successfully the subjects followed instructions to attend to their feelings or perform imagination to the coloured squares. One-sample *t*-test revealed that the subjects’ subjective ratings were significantly higher than chance level [watching: 5.26 vs. 4.00, t(22) = 5.55, *p* < 0.001; implicit: 5.40 vs. 4.00, *t*(22) = 6.34, *p* < 0.001; explicit: 5.02 vs. 4.00, *t*(22) = 4.38, *p* < 0.001, Figure 2C], confirming that subjects followed instructions successfully in watching, explicit, and implicit conditions during the square presentation. Moreover, we examined whether there were differences in the instruction compliance among the watching, implicit, and explicit conditions. One-way ANOVA revealed similar ratings across these three conditions [*F*(2,44) = 1.71, *p* = 0.193]. This suggested that subjects did follow the instructions similarly across the three conditions.

### Emotion Regulation Effects on Subjective Experience

There were no significant differences among watching, explicit, and implicit conditions in experienced fear ratings [*F*(2,44) = 1.13, *p* = 0.33], suggesting that explicit and implicit regulation did not significantly reduce experienced emotions.

### Emotion Regulation Effects in Bilateral Amygdala and Insula

The emotional effects in the bilateral amygdala and insula were analyzed with a repeated measures ANOVA (strategy type: watching, implicit, explicit). A significant main effect of type of strategy [*F*(2,44) = 3.92, *p* = 0.027, η^2^_p_ = 0.151] was observed in the left amygdala but not in the right amygdala [*F*(2,44) = 1.603, *p* = 0.213]. The PSC in the left amygdala was significantly higher in watching than in implicit regulation condition (*p* = 0.009, Figures 3D,E), suggesting that IER by calmness priming was linked with decreased emotion effects in left amygdala activation relative to that for the watching condition. No significant differences were observed when comparing watching and explicit conditions (*p* = 0.29, Figure 3E).

There was a significant main effect of strategy type in left insula activation [*F*(2,44) = 4.80, *p* = 0.013, η^2^_p_ = 0.179]. The PSC was smaller in the implicit than in watching (*p* = 0.006, Figure 4E) or explicit (*p* = 0.045, Figure 4E) conditions in the left insula, and showed a trend toward significance in the right insula (implicit vs. watching, *p* = 0.07, Figure 4F; implicit vs. explicit, *p* = 0.059, Figure 4F). Complementing these results, the direct CS+ vs. CS− comparison during implicit condition was insignificant in bilateral insula (*p* > 0.05, Figure 4D). There were no significant differences between watching and explicit conditions in bilateral insula activity (*p*s > 0.05, Figures 4E,F).

### Cognitive Cost Effects in the Activity of Cognitive Control-Related Regions

**FIGURE 5:**
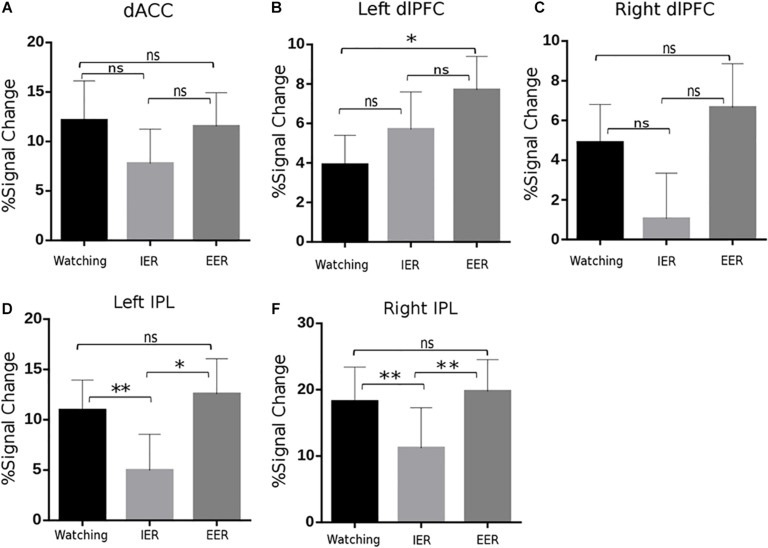
BOLD signal changes in key regions of the frontoparietal control network, including dorsal anterior cingulate cortex (dACC; **A**), bilateral dlPFC **(B,C)**, and IPL **(D,E)** across watching, IER, and EER conditions. It is notable that EER increased activity in the left dlPFC and bilateral IPL compared to that during watching or IER; while IER relative to watching showed similar or reduced activity in these regions. Error bars denote SEM, ^∗^*p* < 0.05, ^∗∗^*p* < 0.01, n.s., not significant.

The activity of cognitive control-related regions was analysed with repeated measures ANOVA (strategy type: watching, implicit, and explicit). A significant main effect of strategy type [*F*(2,44) = 4.01, *p* = 0.025, η^2^_p_ = 0.154] was observed in the left dlPFC and bilateral IPL [left: *F*(2,44) = 7.00, *p* = 0.002, η^2^_p_ = 0.241; right: *F*(2,44) = 8.83, *p* = 0.001, η^2^_p_ = 0.286]. The PSC of the left dlPFC was significantly higher in explicit than in watching conditions (*p* = 0.013, Figure 5B), while there were no significant differences between watching and implicit conditions. For bilateral IPL, the PSC was significantly smaller in implicit than in explicit (left: *p* = 0.012, Figure 5D; right: *p* = 0.008, Figure 5E) and watching conditions (left: *p* = 0.003, Figure 5D; right: *p* < 0.001, Figure 5E). In addition, ANOVA revealed similar activities across watching, explicit, and implicit conditions in the dACC [*F*(2, 44) = 1.04, *p* = 0.36, Figure 5A] and right dlPFC [*F*(2,44) = 2.65, *p* = 0.08, Figure 5C]. These results suggest that explicit regulation by calmness imagination increased cognitive cost (in the left dlPFC), whereas implicit regulation of instructed fear by calmness priming worked without increasing cognitive cost in dACC, bilateral dlPFC, and bilateral IPL activation.

### Task-Related FC Analyses

**FIGURE 6:**
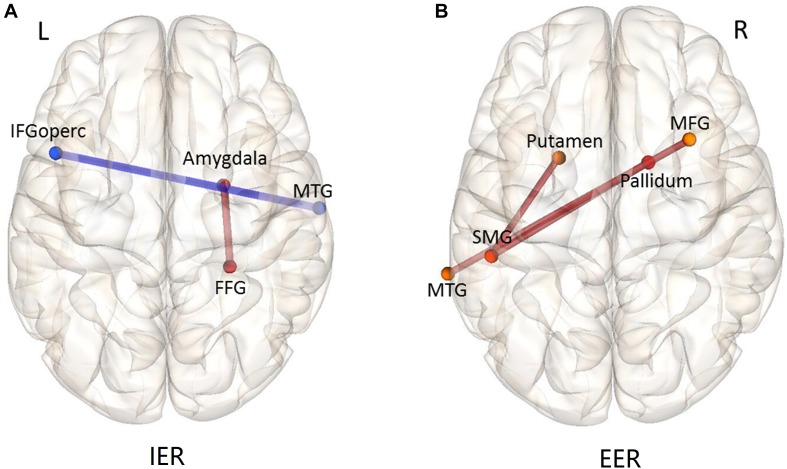
Functional connectivity patterns of the contrast IER **(A)** and EER **(B)** versus watching condition. The connections (edges) between ROIs marked in red show greater FC strength during IER or EER relative to that during watching; those marked in blue show weaker FC strength during IER or EER relative to that during watching. MFG, middle frontal gyrus; IFGoperc, inferior frontal gyrus, opercular part; SMG, supramarginal gyrus; FFG, fusiform gyrus; MTG, middle temporal gyrus.

As stated above, we were mainly interested in the comparison of IER and EER relative to watching conditions, to explore the brain network mechanisms of IER and EER. The results of comparing IER versus watching conditions revealed significantly decreased FC between the right MTG and left IFGoperc, and significantly increased FC between the right amygdala and right fusiform gyrus (see Table 1 and Figure 6A). There were three significantly increased FCs for the contrast of EER versus watching conditions (see Table 1 and Figure 6B), including the FCs between SMG and putamen, SMG and pallidum, and MTG and MFG.

**TABLE 1:** Planned comparisons of functional connectivity strength.

	**FC**	***t*-value**
Implicit vs. watching		
	R MTG - L IFGoperc	−5.05
	R FFG - R Amygdala	5.04
Explicit vs. watching		
	L SMG - L Putamen	4.98
	L SMG - R Pallidum	4.92
	L MTG - R MFG	4.64

*MFG, Middle frontal gyrus; IFGoperc, inferior frontal gyrus, opercular part; SMG, SupraMarginal gyrus; FFG, fusiform gyrus; MTG, middle temporal gyrus.*

## Discussion

The present study examined the emotional consequences, cognitive cost, and neural network bases of IER and EER during the regulation of instructed fear. We observed that IER decreased left amygdala and left insula activity related to the elicitation of instructed fear compared to that for the watching condition, whereas EER failed to show a similar regulatory effect. Moreover, IER did not increase activity across the frontoparietal control network (dACC, bilateral dlPFC) and reduced bilateral inferior parietal lobe activity compared to that for the watching condition. In contrast, EER increased activity of the left dlPFC compared to that for the watching condition. These results suggest that IER by calmness priming reduced neural activation related to instructed fear without increasing cognitive costs, which are often evident during EER.

We did not observe significant regulatory effects of IER on subjective emotional experiences. Previous studies have suggested that IER is more effective at decreasing physiological aspects of negative emotions but is less effective at reducing negative subjective experiences (Williams et al., 2009; Ding et al., 2015; Yuan et al., 2015a). This may be attributed to the subjects’ unawareness of emotion regulation operations. It has also been indicated that the actions of an implicit mind precede the arrival of an explicit mind (Bargh and Morsella, 2008; Damasio, 2012). Thus, IER may decrease physiological reactions first, before its later modulation on subjective experience. The subjective experience and physiological reactions are not synchronized in time because they are considered to be two separate components of emotional episodes (Abelson and Curtis, 1989; Campbell and Ehlert, 2012).

We observed a significant emotion regulation effect of IER in decreasing activity of the left amygdala and left insula. Abundant studies have indicated that the activation of the left amygdala and left insula decreases in successful negative emotion regulation (Phillips et al., 1998; Delgado et al., 2008; Goldin et al., 2008; Diekhof et al., 2011; Giuliani et al., 2011; Kamphausen et al., 2013; Lutz et al., 2013). Specifically, the left amygdala (rather than right amygdala) is particularly involved in the cognitive representation of fear when a stimulus is alerted (Morris et al., 1998) and learnt verbally (Funayama et al., 2001; Phelps et al., 2001; Olsson and Phelps, 2004), and left amygdala activity positively predicts the expression of fear responses measured by skin conductance (Funayama et al., 2001). The reduction in insula activity in response to negative stimuli is also related to a decrease in experienced emotional intensity (Wager et al., 2004, 2011; Schienle et al., 2013). For instance, a decrease in insula activation predicted placebo analgesia (Wager et al., 2011), and placebo eliminated bilateral insula activity for disgust-inducing pictures (Schienle et al., 2013). Considering the important role of the insular cortex in the anticipation of negative events and in conveying cortical representation of fear into the amygdala (Shi and Davis, 1999; Phelps et al., 2001; Herwig et al., 2007a, b; Olsson and Phelps, 2007), we propose that implicit calmness priming may reduce participants’ anticipation of potential threats, consequently leading to reduced subcortical emotional arousal as measured by fear-specific activation in the left amygdala.

IER has been verified to work at little cost of cognitive effort (Gyurak et al., 2011). Thus, its emotion regulation utility should be unaffected by cognitive resource availability. Consistently, we observed decreased activity in the bilateral IPL during IER relative to watching. The IPL is a key node of the dorsal attention network (Zhang and Li, 2014), and is concerned with multiple aspects of sensory processing (Clower et al., 2001; Cabeza et al., 2008; Uncapher and Wagner, 2009) and preparing or applying stimulus selection (Corbetta and Shulman, 2002; Cabeza et al., 2008; Egner et al., 2008; Xu and Chun, 2009). Moreover, the IPL may play a role in the formation of pain memory (Upadhyay et al., 2016). This evidence implies that the implementation of IER should have dulled subjects’ anticipation of instructed threat in the experiment, consequently reducing bilateral IPL activity. Taken together, these findings suggest that IER by calmness priming did not increase the cost of attention and cognitive resources during emotion regulation as measured by dACC, bilateral dlPFC, and bilateral IPL activity.

Concerning EER, we observed no significant reduction in the amygdala and insula but observed higher activity of the left dlPFC during explicit vs. watching conditions. This was consistent with prior findings of increased cognitive demands during intentional emotion regulation (Yuan et al., 2015b). Neuroimaging studies have reported that dlPFC activity increases with cognitive loads, such as increased amount of information held in memory (Braver et al., 1997; Cohen et al., 1997; Jonides et al., 1997; Jansma et al., 2000; Altamura et al., 2007). This is consistent with earlier findings that the implementation of EER increased cognitive costs (Ochsner et al., 2002; Goldin et al., 2008; Hutcherson et al., 2012). However, previous studies reported that guided imagination can reduce the feeling of conditioned fear effectively (Delgado et al., 2008), but we did not replicate this effect in this study. One possible reason is that unlike conditioned fear, instructed fear is an emotional learning response formed by language processing and emotional memory (Olsson and Phelps, 2007). Extensive evidence suggests that instructed fear activates memory-related brain regions (Mechias et al., 2010), and the use of guided imagination also requires memory resources in the meantime. Therefore, a competition for cognitive resources may arise in this case. Another difference between instructed fear and conditioned fear is that the former involves a conscious appraisal of threat, which comprises explicit knowledge of the CS–UCS contingency and consequential cognitive elaborations about the CS and its implications (Mechias et al., 2010). Previous studies have revealed that information about the CS–US contingency prior to fear conditioning enhanced fear responses (Javanbakht et al., 2017). Further, instructed threat may cause anticipated fear or anxiety, which narrows attention and enhances sensitivity to potential danger cues (Cornwell et al., 2011). Since many cognitive resources are occupied by anticipated fear or anxiety, there may be insufficient cognitive resources left for successful imagination of calming situations in the face of fear-conditioned stimuli (Vohs and Heatherton, 2000; Mennin et al., 2005; Shiota and Levenson, 2009). This most likely explains the failure of guided imagination in regulating instructed fear.

FC analysis showed different brain network mechanisms underpinning IER and EER. For the contrast of IER versus watching condition, the intensity of FC between the right fusiform gyrus (FFG) and right amygdala was increased, while the intensity of FC between the middle temporal gyrus (MTG) and opercular part of the inferior frontal gyrus (IFGoperc) was decreased. The fusiform gyrus plays an important role in decoding facial emotions (McCarthy et al., 1997; Kawasaki et al., 2012). Further, Luo et al. (2004) reported that the fusiform gyrus was modulated by emotional valance of words during unconscious repetition priming. Given the important role of FFG in affective priming, the increased FC between the FFG and amygdala identified in this study suggests that IER alleviates fear responses by calmness priming. In addition, prior studies indicate that MTG as a key node of the memory retrieval system (Rolls, 2000) is functionally correlated with the neural networks underpinning controlled retrieval of semantic information, including the inferior frontal gyrus (Davey et al., 2016). In addition, it has been indicated that IFGoperc belongs to the task control network (Power and Petersen, 2013). In this regard, decreased MTG-IFGoperc functional connectivity during IER vs. watching contrast suggests that IER by calmness priming reduced instructed fear-related neural activity without substantial cooperative involvement of control and memory-related networks.

There were three increased FCs during EER than during the watching condition: FCs between the left supramarginal gyrus (SMG) and two nodes (left putamen and right pallidum), and FC between the left MTG and right middle frontal gyrus (MFG). The left SMG plays an important role in the controlled processing of semantic information, such as the operation of verbal working memory and word recognition (Stoeckel et al., 2009; Sliwinska et al., 2012; Deschamps et al., 2014). In addition, the putamen and pallidum are considered motor control networks of the basal ganglia (Robinson et al., 2009). The MTG and MFG have been shown to play an important role in cognitive control networks underpinning task switching (De Baene and Brass, 2013). Taken together, these enhanced FCs that belong to various control systems indicate that the implementation of EER involved more cooperative operations of cognitive control networks, which were absent in the IER condition.

In summary, the current study demonstrated that implicit regulation reduced instructed fear-related neural activity in the left amygdala and left insula, while this emotion regulation effect was absent during explicit regulation. Further, IER did not increase cognitive cost compared to that for the watching condition in key nodes of the frontoparietal control network, while EER increased left dlPFC activity. This favourable effect of implicit regulation on instructed fear was mediated by enhanced FC between the FFG and amygdala, and decreased FC between the MTG and IFGoperc, two neural networks subserving semantic priming and controlled memory selection, respectively. However, this study has the following limitations. Firstly, only Chinese college students participated in this study, hence the result may be lack of representative. Secondly, the duration of EER for threat was relatively short and consistent in current study. Previous studies have shown that the duration of exposure plays an important role in systematic desensitization (Watts, 1979). The duration time should be considered as a factor of explicit regulation in further study when compared with implicit regulation.

## Data Availability Statement

The datasets generated for this study are available on request to the corresponding author.

## Ethics Statement

The studies involving human participants were reviewed and approved by the local ethical committee of the Southwest University for human brain research. The patients/participants provided their written informed consent to participate in this study. Written informed consent was obtained from the individual(s) for the publication of any potentially identifiable images or data included in this article.

## Author Contributions

YZ and SC completed the data analysis and wrote the manuscript. ZD conducted the experiments and performed the data analysis. JYa assisted in the experimental operation. JYu proposed the study, wrote the manuscript, and supervised all the research. All authors approved the final version of the manuscript.

## Conflict of Interest

The authors declare that the research was conducted in the absence of any commercial or financial relationships that could be construed as a potential conflict of interest.

## Footnotes

1 www.fil.ion.ucl.ac.uk/SPM

2 www.nitrc.org/projects/conn
